# Circulating plasma lncRNAs as novel markers of *EGFR* mutation status and monitors of epidermal growth factor receptor‐tyrosine kinase inhibitor therapy

**DOI:** 10.1111/1759-7714.13216

**Published:** 2019-11-05

**Authors:** Panpan Lv, Shaoxing Yang, Wenjing Liu, Haifeng Qin, Xiuhua Tang, Fangfang Wu, Zeyuan Liu, Hongjun Gao, Xiaoqing Liu

**Affiliations:** ^1^ Academy of Military Medical Science Beijing China; ^2^ PLA Rocket Force Characteristic Medical Center Beijing China; ^3^ Department of Pulmonary Oncology The Fifth Medical Centre, Chinese PLA General Hospital Beijing China

**Keywords:** Biomarker, epidermal growth factor receptor, long noncoding RNA, non‐small cell lung cancer, tyrosine kinase inhibitor

## Abstract

**Background:**

Epidermal growth factor receptor (EGFR) gene mutations predict tumor response to EGFR tyrosine kinase inhibitors (EGFR‐TKIs) in non‐small cell lung cancer (NSCLC). However, even patients with *EGFR*‐sensitive mutations in NSCLC have limited efficacy with EGFR‐TKI. Studies have shown that long noncoding RNA (lncRNA) is related to diagnosis and prognosis with NSCLC. This study aimed to explore the correlation between lncRNA in NSCLC patients with *EGFR* mutation status and EGFR‐TKI efficacy.

**Methods:**

The amplification‐refractory mutation system method was used to test the *EGFR* mutation status in tumor tissues and pleural effusions of NSCLC patients. Three *EGFR*‐mutant patients and three EGFR wild‐type patients were selected. Differential lncRNA was performed on the pleural effusions of the two selected groups of patients using Clariom D Human chip technology. Five lncRNAs significantly associated with *EGFR* mutation status were screened by FC value and GO analysis, and then evaluated by real‐time quantitative polymerase chain reaction in NSCLC patients' pleural effusions. Three were further analyzed in NSCLC patients' plasma.

**Results:**

There were 61 significant differences in lncRNA between *EGFR* mutation‐positive and wild‐type patients. Among them, SCARNA7, MALAT1, NONHSAT017369, NONHSAT051892, and FTH1P2 were significantly associated with *EGFR* mutation status. SCARNA7, MALAT1, and NONHSAT017369 showed consistent results with plasma in pleural effusions compared to EGFR wild‐type, all upregulated in the *EGFR* mutation group.

**Conclusion:**

This study shows that lncRNAs can be used not only as potential biomarkers for predicting the mutation status of *EGFR a*nd the efficacy of EGFR‐TKI, but also for monitoring the efficacy of EGFR‐TKI.

## Introduction

As one of the cancers with the highest morbidity, lung cancer ranks first among all tumors worldwide in terms of mortality.^1^ Non‐small cell lung cancer (NSCLC) accounts for approximately 80.0%–85.0% of all lung cancer cases.^2^, ^3^ Most NSCLC patients, especially those receiving traditional chemotherapy, are at an advanced stage at the time of diagnosis and have a poorer prognosis. According to the National Comprehensive Cancer Network (NCCN) guidelines, patients with *EGFR*‐mutant disease (mainly the *EGFR* exon 19 deletion and p.L858R point mutation) are advised to undergo EGFR‐tyrosine kinase inhibitor (TKI) therapy as first‐line treatment.^4^ It is therefore important to identify the tumor genotype after histopathological determination.

Clinically, obtaining sufficient tumor specimens for pathological classification and genotyping is the decisive condition for establishing personalized treatment for patients. However, tumor tissue specimens cannot be obtained from some patients. Moreover, rebiopsy during EGFR‐TKI therapy in order to continuously monitor *EGFR* mutation status and analyze EGFR‐TKI resistance remains a major challenge. In recent years, liquid biopsy has gained extensive attention as a noninvasive detection method. Its unique advantage is that it overcomes tumor heterogeneity to some extent, which can help clinicians adjust the treatment strategy for NSCLC patients in a timely manner. In fact, circulating tumor DNA (ctDNA) demonstrates high accuracy in the analysis of *EGFR* mutation status, but its sensitivity is relatively low.^3^, ^5^, ^6^, ^7^ The sensitivity of ctDNA ranges between 70.0% and 75.0%, and a possible explanation is that the ability to detect gene mutations in plasma ctDNA is related to tumor load; for patients with a smaller tumor load, the false‐negative rate will increase significantly. Another possible reason is that somatic mutations accumulated by aging cells restrict the utility of ctDNA.^7^, ^8^ At the same time, a number of studies have shown that the efficacy of EGFR‐TKI in patients with *EGFR*‐mutant NSCLC is only about 70%, which may be related to the activation of the bypass signaling pathway to cause primary resistance.^9^, ^10^, ^11^ However, there is currently no clinically relevant marker to predict the efficacy of EGFR‐TKI. Therefore, it is necessary to define a group of noninvasive, convenient, economical markers as a supplement for predicting *EGFR* mutation status and monitoring the efficacy of EGFR‐TKI therapy in patients with NSCLC.

In previous studies, more attention was given to the relationship of the protein coding gene mutation with the tumor. With the continuous understanding of lncRNA, it is believed that the differential expression and mutation of lncRNA play an important role in the malignant proliferation of tumors. Over the past decade, lncRNA has been found to be stably expressed in human blood, and circulating lncRNAs can serve as novel molecular biomarkers for diseases and cancers.^12^, ^13^, ^14^ Blood biomarkers have several significant advantages: they are easily obtained in a minimally invasive manner, can be measured repeatedly, and their expression levels can be compared longitudinally. However, the association between circulating plasma lncRNAs and *EGFR* mutation status and the role of lncRNAs in monitoring the efficacy of EGFR‐TKI therapy have yet to be systematically studied. Our objective was to identify a group of circulating plasma lncRNAs that can predict *EGFR* mutation status and explore the potential of this group of lncRNAs to monitor the efficacy of EGFR‐TKI therapy.

Compared to other chip technologies, Clariom D solutions offer unprecedented comprehensive transcriptome coverage, providing deeper, broader, and more sophisticated transcriptome information detection. Samples for different sources and properties, including whole blood and pleural effusion samples, are the tools of choice for transformation studies. A single experiment can accurately detect the difference in expression of coding RNA and noncoding RNA at both gene and exon levels, as well as enriching the comprehensive variable shear information. Plasma and pleural effusion samples were used in this study. A number of studies have shown that pleural effusions are more sensitive to detecting *EGFR* mutation status than blood samples. To improve the specificity and sensitivity of this study, we used Clariom D Human Chip technology to detect pleural effusions with different *EGFR* mutation status.

## Methods

### Patient enrollment

This study was approved by the Ethics Committee of the Fifth Medical Center of the Chinese PLA General Hospital (No. 2012‐11‐171). All experiments were carried out in accordance with the Declaration of Helsinki. From December 2016 to April 2018, patients with NSCLC (*n* = 155) were recruited from the Department of Pulmonary Oncology of the Fifth Medical Center of the Chinese PLA General Hospital. Informed consent was obtained from all patients. Inclusion criteria for patients were as follows: (i) NSCLC diagnosis by histopathology or cytology (stage IIIb, IV); (ii) age ≥ 18 years old; (iii) Eastern Cooperative Oncology Group Performance Status, (ECOG PS) <2 points; (iv) no prior chemotherapy, radiotherapy, targeted therapy, and other oncology treatment; and (v) *EGFR* gene detection by histology. Patients with diseases of the heart, liver, kidney, and other important organs were excluded. The study flow chart is shown in Figure S1.

### Plasma (pleural effusion) collection

A total of 5 mL of peripheral blood was collected between 0700 and 0800 hours (15 mL of pleural effusion was collected during thoracentesis). Blood samples were dynamically collected from 36 of the 72 patients with *EGFR* mutations at one, three, and five months of EGFR‐TKI treatment. In addition, blood samples were collected from 16 patients at the time of progressive disease (PD). Plasma (pleural effusion) was separated by centrifugation at 4500 ***g*** at 4°C for 10 minutes within 30 minutes after collection and immediately stored at −80°C for further analysis. QIAzol lysis reagent (Cat. No. 217184; QIAGEN Inc., Valencia, CA, USA) was used to separate total RNA containing lncRNA from 200 μL of plasma (pleural effusion).

### Analysis of lncRNA in pleural effusions

Clariom D Human chip (Guangdong Geneway Decoding Bio‐Tech Co. Ltd., Guangzhou, China) analysis was performed on the pleural effusions from three *EGFR*‐mutant patients and three EGFR wild‐type patients with NSCLC to initially screen for lncRNAs that were differentially expressed in these patients.

### qRT‐PCR of selected lncRNAs

Reverse transcription was performed using the All‐in‐One cDNA Synthesis SuperMix kit (Bimake, Houston, USA) according to the manufacturer's instructions. Moreover, 2× SYBR Green qPCR Master Mix kit (QIAGEN) was used for the qRT‐PCR analysis of the expression levels of the selected lncRNAs in pleural effusions and plasma. PCR was performed in a total reaction volume of 23 μL, including 10 μL 2× SYBR Green qPCR Master Mix, 5 μL cDNA template, 2 μL forward primer (5 mM), 2 μL reverse primer (5 mM), 0.4 μL ROX Reference Dye, and 3.6 μL deionized water. qRT‐PCR was performed in triplicate using the PCR program of the Applied Biosystems 7500 PCR System (Applied Biosystems, Thermo Fisher Scientific Corp. 5, USA). Denaturation was performed at 95.0°C for 10 minutes, followed by 45 cycles of denaturation at 95.0°C for 15 seconds, 60.0°C for 30 seconds, and 72.0°C for 30 seconds. The original Ct data was normalized to the expression level of 18S rRNA using the 2^−Δ(ΔCt)^ method.

### Statistical analysis

Statistical analysis was performed using SPSS 17.0 (IBM Corp., Armonk, NY, USA). Student's *t*‐test was used to evaluate differences in the expression of the chosen lncRNAs in pleural effusion and plasma from the NSCLC patients. The specificity, sensitivity, and area under the curve (AUC) for the lncRNA levels were determined using ROC analysis. Using the binary outcome of *EGFR*(+) and *EGFR*(−) samples as dependent variables, a logistic regression model was established using the stepwise model selection method. All statistical tests were two‐tailed, and a *P*‐value < 0.05 was considered statistically significant.

## Results

### Patient characteristics

From December 2016 to April 2018, a total of 490 patients were included with advanced NSCLC confirmed by tissue or cytopathology with a median age of 58.2 (range 37–82) years and a male‐to‐female ratio of 1:0.74. According to the inclusion criteria, we selected 77 patients with *EGFR* mutation status confirmed in the tumor tissue by histological examination from 218 patients with NSCLC with pleural effusion, and those diagnosed with tumor cells in pleural effusion for the study (35 *EGFR* mutants, 42 EGFR wild‐type). The clinical features of EGFR wild‐type patients are summarized in Table 1. A number of studies have shown that the specificity and sensitivity of ctDNA with EGFR mutation status detected in pleural effusion are close to, or even higher than, the tumor tissue.^15^, ^16^ Among 372 patients with *EGFR* mutation status confirmed by tumor tissue, 142 patients who did not receive any lung cancer treatment were enrolled in the study (72 *EGFR* mutants, 70 EGFR wild‐type), and their clinical features are summarized in Table 1. No significant differences in clinical features were observed between the *EGFR*‐mutant group and the EGFR wild‐type group, except for their smoking status; there were fewer smokers in the *EGFR*‐mutant group (*P* < 0.05).

**Table 1:** Clinicopathological characteristics of non‐small cell lung carcinoma patients based on epidermal growth factor receptor mutation status at the time of primary diagnosis

	Plasma			Pleural effusion		
Characteristic	_*EGFR*_MUT(+) (*n* = 72)	*EGFR* ^WT^ (*n* = 70)	*P*‐value	_*EGFR*_MUT(+) (*n* = 35)	*EGFR* ^WT^ (*n* = 42)	*P‐*value
Age (years), mean (range)	53.9 (32–80)	57.0 (36–82)	N/A	48.0 (31–74)	52.1 (36–79)	
Gender, *n* (%)			0.093			0.386
Male	30 (41.7%)	41 (58.6%)		16 (45.7%)	29 (69.0%)	
Female	42 (58.3%)	29 (41.4%)		19 (54.3%)	13 (31.0%)	
ECOG PS, *n* (%)			N/A			N/A
0	4 (5.6%)	2 (2.9%)		3 (8.6%)	2 (4.8%)	
1	58 (94.4%)	68 (97.1%)		31(88.6%)	40 (95.2%)	
2	0 (0.0)	0 (0.0)		1(2.9%)	0 (0.0)	
Smoker, *n* (%)			0.010*			0.001*
Never smokers	51 (70.8%)	32 (45.7%)		25 (71.4%)	11 (26.2%)	
Smokers	21 (29.2%)	38 (54.3%)		10 (28.6%)	31 (73.8%)	
cStage, *n* (%)			0.900			0.710
IIIA–B	6 (8.3%)	7 (10.0%)		4 (11.4%)	6 (14.3%)	
IV	66 (91.7%)	63 (90.0%)		31 (88.6%)	36 (85.7%)	
Histologic subtype, *n* (%)			0.633			0.191
Adenocarcinoma	70 (97.2%)	69 (98.6%)		35 (100%)	40 (95.2%)	
Squamous carcinoma	0 (0.0)	0 (0.0)		0	0	
Other	2 (2.8%)	1(1.4%)		0	2 (4.8%)	
Family history of cancer, *n* (%)			0.768			0.774
Y	14 (19.4%)	13 (18.6%)		5 (14.3%)	7(16.7%)	
N	58 (80.6%)	57 (81.4%)		30 (85.7%)	35 (83.3%)	

* *P* < 0.05.

19DEL, exon 19 deletion; ADC, adenocarcinoma; cStage, clinical stage; ECOG PS, Eastern Cooperative Oncology Group performance status; EGFR, epidermal growth factor receptor; F, female; FH, family history; M, male; MUT(+), mutation‐positive; N, no; N/A, not applicable; NSCLC, non‐small cell lung cancer; SCC, squamous cell carcinoma; WT, wild‐type; Y, yes.

### Efficacy analysis

Of the 72 patients with advanced NSCLC *EGFR* mutations, 68 received a first‐generation EGFR‐TKI treatment. Among them, 58 patients (58/68, 85.3%) received icotinib, eight received (8/68, 11.8%) gefitinib, and two received (2/68, 2.9%) erlotinib. A total of 59 patients with EGFR‐TKI treatment were followed‐up until December 2018 for disease progression. Among these patients, first‐line evaluation revealed: complete response (CR), 0 cases; partial response (PR), 34 cases (34/59, 57.6%); SD, 14 cases (14/59, 23.7%); PD, 11 (11/59, 18.6%); and mean progression‐free survival for 10 months (range, 1–24).

### Gene chip analysis of candidate lncRNAs associated with *EGFR* mutation

**Figure 1:**
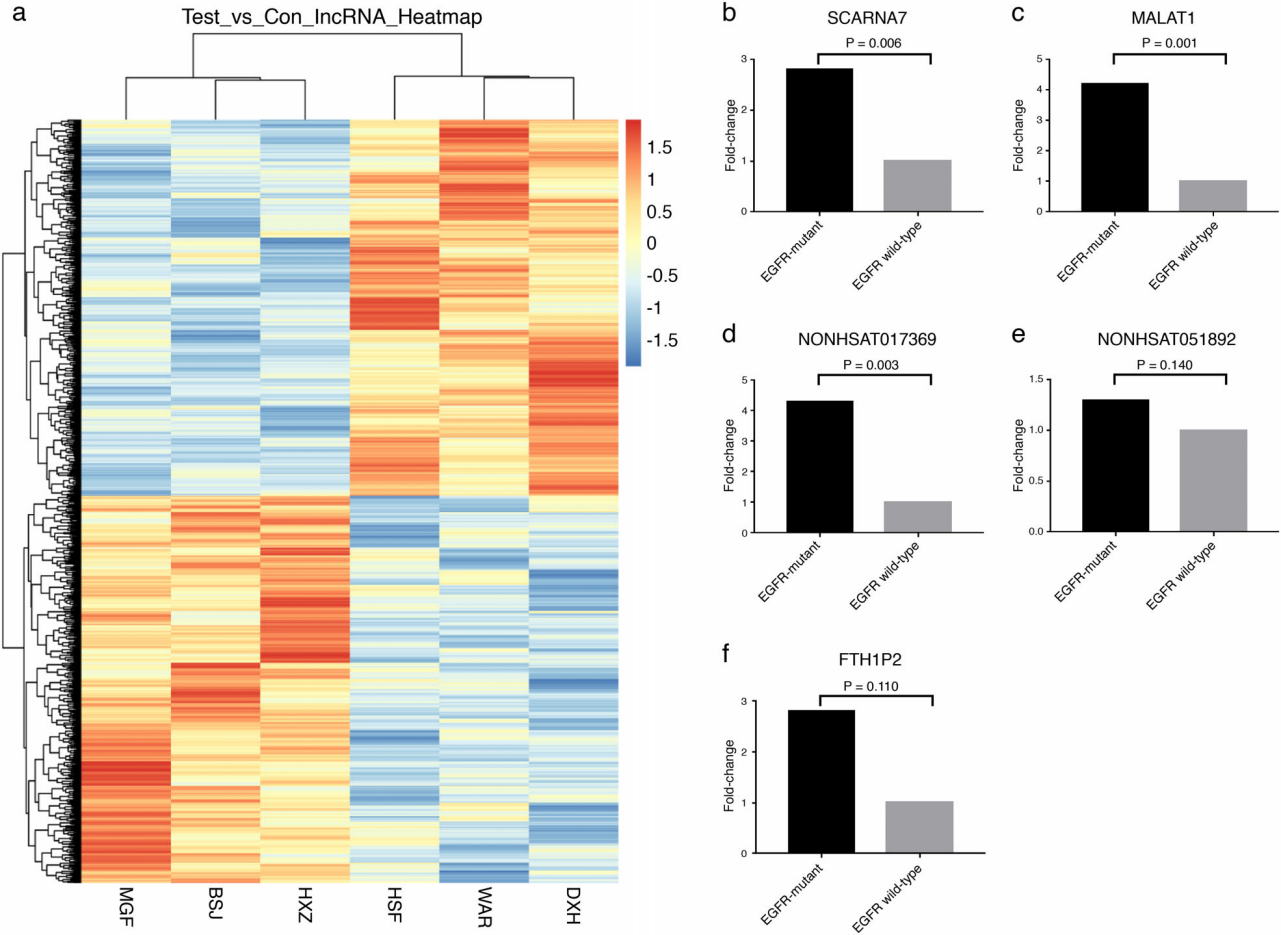
(**a**) Cluster analysis of lncRNA expression profiles in pleural effusion from EGFR‐positive NSCLC, EGFR‐negative NSCLC. MGF, BSJ, and HXZ represent the EGFR‐positive. HSF, WAR, and DXH represent the EGFR‐negative. Experimental analysis of the differential expression of five selected lncRNAs detected in pleural effusions of *EGFR*‐mutant versus wild‐type NSCLC patients (**b**) SCARNA7, (**c**) MALAT1, (**d**) NONHSAT017369, (**e**) NONHSAT051892, and (**f**) FTH1P2. EGFR, epidermal growth factor receptor; lncRNA, long noncoding RNA; N/A, not applicable; NSCLC, non‐small cell lung cancer.

Pleural effusion samples of three *EGFR*‐mutant and three EGFR wild‐type patients were selected for Clariom D Human microarray analysis to detect the differences in the expression of lncRNAs (Fig 1a). Compared with the EGFR wild‐type group, 48 lncRNAs were upregulated and 13 lncRNAs downregulated in the *EGFR*‐mutant group (fold change ≥2 or ≤0.5, *P* < 0.05; Table 2). Five of these lncRNAs were selected for further validation. Among the five lncRNAs, *SCARNA7*, *MALAT1*, *NONHSAT017369*, and *NONHSAT051892* were significantly upregulated and *FTH1P2* was significantly downregulated in *EGFR*‐mutant patients.

**Table 2:** Microarray analysis of the differential expression of long noncoding RNA in patients with epidermal growth factor receptor‐mutant versus epidermal growth factor receptor‐wild‐type non‐small cell lung carcinoma

LncRNA	Fold‐change	Regulation	*P*‐value
*SCARNA7*	12.41	Up	0.0083
*MALAT1*	9.71	Up	0.0086
*NONHSNONHSAT017369*	7.31	Up	0.0087
*NONHSAT051892*	5.98	Up	0.0075
*SCARNA8*	5.58	Up	0.0006
*SCARNA18*	4.96	Up	0.0076
*NONHSAT097341*	4.65	Up	0.0094
*GUSBP2*	4.58	Up	0.0140
*NONHSAT051297*	4.40	Up	0.0323
*NONHSAT051221*	3.80	Up	0.0164
*NONHSAT040380*	3.68	Up	0.0012
*NONHSAT102434*	3.65	Up	0.0003
*NONHSAT051251*	3.53	Up	0.0480
*NONHSAT131588*	3.52	Up	0.0281
*NONHSAT140284*	3.18	Up	0.0245
*NONHSAT140294*	3.12	Up	0.0212
*NONHSAT142099*	3.02	Up	0.0001
*WASH2P*	2.81	Up	0.0330
*NONHSAT140289*	2.81	Up	0.0466
*NONHSAT140280*	2.62	Up	0.0366
*NONHSAT040416*	2.59	Up	0.0332
*SCARNA17*	2.58	Up	0.0029
*NEAT1*	2.58	Up	0.0337
*GUSBP9*	2.48	Up	0.0366
*HMGN2P10*	2.47	Up	0.0062
*SCARNA5*	2.43	Up	0.0311
*STAG3L2*	2.37	Up	0.0354
*NONHSAT094176*	2.32	Up	0.0151
*lnc‐PRKRIP1‐1:1*	2.29	Up	0.0011
*NONHSAT113557*	2.26	Up	0.0246
*NONHSAT112993*	2.24	Up	0.0215
*NONHSAT010383*	2.23	Up	0.0002
*ATP6V1G2‐DDX39B*	2.19	Up	0.0429
*PRR20FP*	2.18	Up	0.0346
*NONHSAT044166*	2.17	Up	0.0141
*NONHSAT009235*	2.14	Up	0.0186
*lnc‐IL32‐1:1*	2.13	Up	0.0097
*T259406*	2.13	Up	0.0018
*DNM1P46*	2.10	Up	0.0309
*RP11‐632K20.8*	2.09	Up	0.0495
*LRRC37A*	2.09	Up	0.0153
*NONHSAT093401*	2.09	Up	0.0288
*NONHSAT060570*	2.08	Up	0.0039
*NONHSAT133451*	2.07	Up	0.0427
*BRD9P2*	2.06	Up	0.0496
*RP11‐351I24.1*	2.01	Up	0.0323
*AC104451.2*	2.00	Up	0.0254
*RP11‐274B21.12*	2.00	Up	0.0215
*CTC‐258N23.3*	−2.09	Down	0.0203
*NONHSAT017039*	−2.16	Down	0.0042
*CTD‐2014B16.2*	−2.26	Down	0.0342
*FTH1P12*	−2.33	Down	0.0249
*NONHSAT100900*	−2.33	Down	0.0013
*FTH1P16*	−2.70	Down	0.0427
*FTH1P4*	−2.71	Down	0.0239
*FTH1P20*	−2.76	Down	0.0285
*FTH1P11*	−3.13	Down	0.0154
*FTH1P23*	−3.15	Down	0.0456
*VTRNA1‐1*	−3.63	Down	0.0438
*FTH1P10*	−3.94	Down	0.0155
*FTH1P2*	−4.09	Down	0.0154

lncRNA, long noncoding RNA.

### qRT‐PCR validation of candidate lncRNAs

Based on the results of the pleural effusion chip, real‐time quantitative polymerase chain reaction (qRT‐PCR) was used to validate the expression of the five lncRNAs in the pleural effusions in 35 *EGFR*‐mutant and 42 EGFR wild‐type patients. The 18S ribosomal RNA (18S rRNA) was used as an internal control to normalize the original cycle quantification (Ct) values. The Ct of 18S rRNA was subtracted from the Ct value of the target lncRNA (ΔCt) and converted to 2^−ΔCt^ to evaluate the changes in the lncRNA expression between the groups. The fold change was calculated using the 2^−Δ(ΔCt)^ method, where Δ(ΔCt) = (Ct LncR − Ct 18S rRNA) *EGFR*‐mutant − (Ct LncR − Ct 18S rRNA) EGFR wild‐type. Figure 1b–f show the relative expression levels of the five lncRNAs in the *EGFR*‐mutant group and the EGFR wild‐type group. The expression levels of both *NONHSAT051892* and *FTH1P2* were not significantly different between the *EGFR*‐mutant and wild‐type groups. Therefore, *SCARNA7*, *MALAT1*, and *NONHSAT017369* were selected for further study.

### Circulating plasma lncRNA for predicting *EGFR* mutation

**Figure 2:**
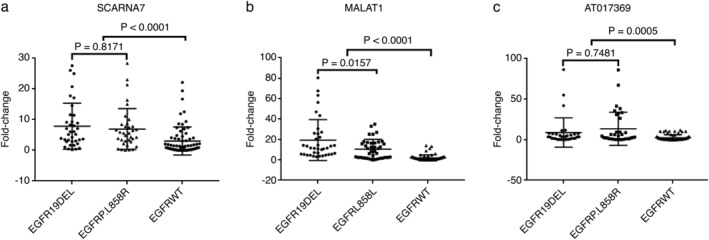
Plasma levels of SCARNA7 (**a**), MALAT1 (**b**), and NONHSAT017369 (**c**) are higher in patients with EGFR‐positive (EGFR19DEL and EGFRP.L858R) non‐small cell lung cancer. The lncRNA expression levels in the EGFR‐positive and EGFR‐negative non‐small cell lung cancer cohorts (x‐axis) are shown as 2^−ΔΔCt^ values (y‐axis), as calculated from real‐time quantitative polymerase chain reaction.

To further investigate the circulating plasma lncRNA in *EGFR*‐mutant patients with NSCLC, qRT‐PCR analysis of the plasma of *EGFR*‐mutant and EGFR wild‐type patients was performed using 18S rRNA as an internal control. We observed the same trend as we did in the pleural effusions, namely, significant upregulation of *SCARNA7*, *MALAT1*, and *NONHSAT017369* in the *EGFR*‐mutant group compared to the EGFR wild‐type group (*P* < 0.05; Fig 2). Further analysis of patients harboring *EGFR* exon 19 deletions and p.L858R point mutations revealed that *MALAT1* was upregulated in the *EGFR* exon 19 deletion group compared to the *EGFR* p.L858R point mutation group (*P* < 0.05; Fig 2); no statistically significant differences were observed in the expression of the remaining two lncRNAs by *EGFR* mutation.

### Diagnostic analysis of *EGFR* mutation status by detection of circulating plasma lncRNA in patients with NSCLC

**Figure 3:**
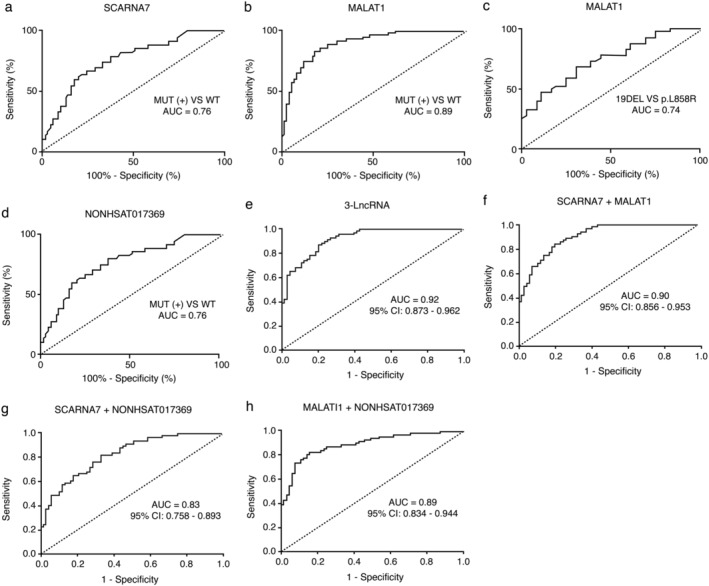
ROC curve analyses demonstrated that plasma levels of SCARNA7, MALAT1, and NONHSAT017369 differed between patients with EGFR positive and EGFR negative NSCLC. (**a**, **b** and **d**) ROC curves showing that plasma levels of SCARNA7, MALAT1, and NONHSAT017369 differ between patients with EGFR‐positive and EGFR‐negative NSCLC. (**c**) ROC curve analyses of the difference between MALAT1 with EGFR19DEL and EGFRpL858R. (**e**) Combination ROC curve analyses of the 3 lncRNAs as a means of distinguishing between patients with EGFR‐positive and EGFR‐negative NSCLC. The AUC is higher the two combination: (**f**) SCARNA7+MALAT1, (**g**) SCARNA7+NONHSAT017369, and (**h**) MALAT1+NONHSAT017369. AUC, area under the curve; ROC, receiver operating characteristic.

The potential of circulating plasma *SCARNA7*, *MALAT1*, and *NONHSAT017369* as diagnostic markers for *EGFR* mutation status in NSCLC patients was evaluated using ROC curve analysis. Circulating *SCARNA7* was significantly different between *EGFR*‐mutant patients and EGFR wild‐type patients, with a ROC‐AUC of 0.76 (95% confidence interval [CI]: 0.68–0.84), sensitivity of 80.8%, and specificity of 67.1% (Fig 3a). No significant differences were found between the *EGFR* exon 19 deletion group and the *EGFR* p.L858R point mutation group. Circulating *MALAT1* was also significantly different between *EGFR*‐mutant patients and EGFR wild‐type patients, with a ROC‐AUC of 0.89 (95% CI: 0.84–0.95), sensitivity of 79.2%, and specificity of 85.7% (Fig 3b). *MALAT1* was significantly upregulated in the *EGFR* exon 19 deletion group compared to the *EGFR* p.L858R point mutation group, with a ROC‐AUC of 0.74 (95% CI: 0.64–0.85), sensitivity of 70.0%, and specificity of 69.0% (Fig 3c). *NONHSAT017369* could only distinguish between the *EGFR*‐mutant group and the EGFR wild‐type group. The ROC‐AUC was 0.76 (95.0% CI: 0.68–0.84), the sensitivity was 79.7%, and the specificity 63.6% (Fig 3d). To further evaluate the diagnostic potential of the combinations of *SCARNA7*, *MALAT1*, and *NONHSAT017369*, a logistic regression analysis was conducted to combine the ROC curve analyses of these three lncRNAs. The AUC in the 3‐lncRNA group was 0.920 (95% CI: 0.860–0.981) (Fig 3e). Furthermore, the characteristics of 2‐lncRNA groups were examined (Fig 3f–h). Two lncRNAs were randomly selected from the three lncRNAs, and the AUC of all the combinations was lower than that in the 3‐lncRNA group. The positive predictive value (PPV) and negative predictive value (NPV) of each model were also calculated to distinguish between *EGFR*‐mutant patients and EGFR wild‐type patients. As shown in Table 3, the PPV of the three individual lncRNAs was higher than 68.5%, and the NPV was higher than 74.5%. The combination of the three lncRNAs could distinguish between the *EGFR*‐mutant patients and the EGFR wild‐type patients, and the PPV and NPV were both higher than 80%. Overall, the results indicate that the plasma levels of the three lncRNAs have a high diagnostic value for distinguishing between *EGFR*‐mutant and EGFR wild‐type patients.

**Table 3:** Measures of diagnostic performance for distinguishing patients with epidermal growth factor receptor‐mutant and epidermal growth factor receptor‐wild‐type non‐small cell lung cancer

Variables	*SCARNA7*	*MALAT1*	*NONHSAT017369*	3‐lncRNA
Cut‐off	2.105	3.448	1.209	0.334
Sensitivity	80.8%	79.2%	79.7%	87.0%
Specificity	67.1%	85.7%	63.6%	79.4%
PPV	72.0%	88.4%	68.8%	82.2%
NPV	77.0%	77.5%	74.5%	84.7%

3‐lncRNA includes *SCARNA7*+*MALAT1*+*NONHSAT017369*.

lncRNA, long noncoding RNA; NPV, negative predictive value; PPV, positive predictive value.

### Relationship between the initial level of circulating plasma lncRNAs and progression‐free survival in patients treated with EGFR‐TKIs

**Figure 4:**
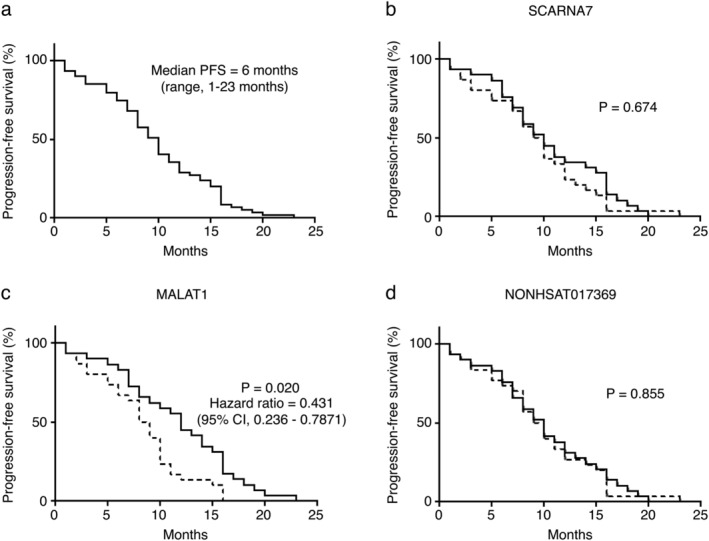
The relationship between the initial expression levels of plasma lncRNAs and the progression‐free survival (PFS) of EGFR‐positive patients receiving EGFR‐TKI (n = 59). (**a**) PFS of EGFR‐positive patients who received EGFR‐TKI therapy. (**b**) PFS of EGFR‐positive patients, stratified according to the expression levels of SCARNA7. (**c**) PFS of EGFR‐positive patients, stratified according to the expression levels of MALAT1. (**d**) PFS of EGFR‐positive patients, stratified according to the expression levels of NONHSAT017369. All survival rates were estimated using the Kaplan‐Meier method. (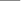) High expression and (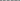) low expression.

Of the 72 *EGFR*‐mutant patients with NSCLC, 68 received EGFR‐TKI therapy, and 59 developed resistance to EGFR‐TKIs and displayed disease progression. Kaplan‐Meier analysis was performed for progression‐free survival (PFS) based on clinicopathological factors, as well as the initial (before EGFR‐TKI) plasma levels of *SCARNA7*, *MALAT1*, and *NONHSAT017369*. As 55 of the 59 patients had an Eastern Cooperative Oncology Group (ECOG) score of one, and only four patients had an ECOG score of 0, we did not perform Kaplan‐Meier analysis based on ECOG score. The plasma expression levels of the three lncRNAs were stratified by the median value. The PFS of patients with high lncRNA expression levels (≥the median) was compared to that of patients with low lncRNA expression levels (<the median). As shown in Figure 4, the median PFS of the 59 patients was 10 months (range: 1–24 months). The initial expression of *SCARNA7* and *NONHSAT017369* had no significant correlation with PFS (*P* = 0.67 and *P* = 0.855, respectively). However, the pretreatment expression of *MALAT1* was significantly correlated with PFS (*P* = 0.020). In patients with high and low expression of *MALAT1*, the median PFS were 11.6 and 8.2 months, respectively (hazard ratio: 0.431; 95% CI: 0.236–0.787). As indicated in Table 4, multifactor analysis showed smoking history was also significantly correlated with PFS (hazard ratio: 2.33; 95% CI, 1.28–4.27; *P* = 0.006). These results indicated that both *MALAT1* and smoking history were important predictors of the efficacy of EGFR‐TKIs in patients with NSCLC.

**Table 4:** Univariate and multivariate analyses of clinicopathological variables associated with progression‐free survival

Clinical variables	Hazard ratio (95% CI)	*P‐*value
Univariate analysis		
Gender (male vs. female)	1.32 (0.78–2.240)	0.244
Age (≥60 years vs. <60 years)	1.03 (0.61–1.75)	0.896
Stage (IIIB vs. IV)	0.63 (0.31–1.276)	0.205
Smoking history (never smokers vs. smokers)	2.28 (1.18–4.42)	0.001
SCARNA7 (low‐expression vs. high‐expression)	0.82 (0.49–1.37)	0.412
MALAT1 (low‐expression vs. high‐expression)	0.54 (0.32–0.928)	0.020
NONHSAT017369 (low‐expression vs. high‐expression)	0.96 (0.58–1.60)	0.855
Multivariate analysis		
Smoking history (never smokers vs. smokers)	2.33 (1.28–4.27)	0.006
MALAT1 (low‐expression vs. high‐expression)	1.84 (1.06–3.21)	0.032

CI, confidence interval. In the univariate analysis the progression‐free survival was analyzed using Kaplan‐Meier method, and hazard ratios were estimated using log rank test. In multivariate analysis, hazard ratios were estimated using the multivariate Cox proportional hazards regression model.

### Circulating plasma lncRNAs as a potential marker for monitoring the efficacy of EGFR‐TKI therapy

**Figure 5:**
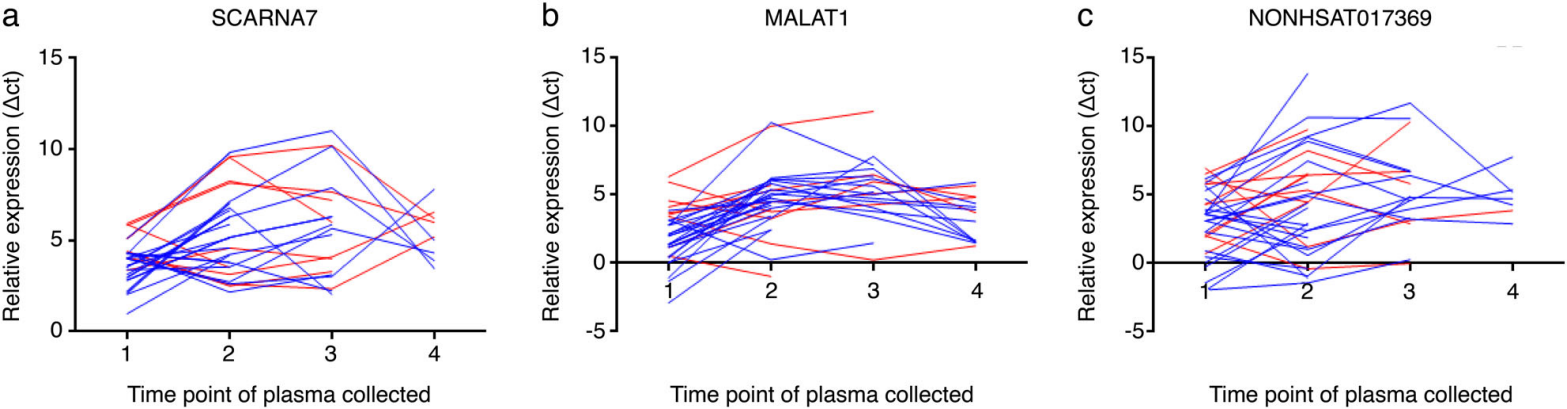
The changes of plasma levels of SCARNA7, MALAT1, and NONHSAT017369 before and after treatment with EGFR‐TKI. The y‐axes show the fold change of SCARNA7 (**a**), MALAT1 (**b**), and NONHSAT017369 (**c**) which were normalized to 18S rRNA. EGFR‐TKI, epidermal growth factor receptor‐tyrosine kinase inhibitor. (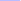) PR and (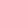) SD.

To determine whether the plasma levels of *SCARNA7*, *MALAT1*, and *NONHSAT017369* had predictive value for the efficacy of EGFR‐TKI therapy, qRT‐PCR was used to compare the plasma levels of the three lncRNAs in 36 patients before and after EGFR‐TKI treatment. A total of 78 plasma samples were collected from the 36 patients before and after EGFR‐TKI treatment. Initial blood samples were collected two weeks (± two days) before the treatment and then every two months (± one week). At the same time, chest computed tomography (CT) was performed to evaluate the response to treatment. Of the 36 patients, 23 achieved PR, nine had SD, and four had PD. The plasma lncRNA levels before and after treatment are shown in Figure 5, and they were analyzed by Wilcoxon matched pairs signed rank sum test. Of the 32 patients achieving PR and SD after EGFR‐TKI treatment, 27 exhibited decreased plasma *MALAT1* levels (*P* < 0.05), and 23 displayed decreased *SCARNA7* levels (*P* < 0.05) after treatment. However, no similar trend was observed in patients with PD. There was no statistically significant difference in the plasma levels of *NONHSAT017369* after EGFR‐TKI treatment (*P* > 0.05). Overall, the study on the three lncRNAs showed that the changes in the plasma levels of *SCARNA7* and *MALAT1* from before to after EGFR‐TKI treatment had a satisfactory predictive value regarding the efficacy of EGFR‐TKI therapy.

## Discussion

The assessment of the *EGFR* gene mutation status in lung cancer tissues has important predictive value for the efficacy of EGFR‐TKI. However, lung cancer tissue in patients with advanced NSCLC is sometimes difficult to obtain. Although clinically circulating ctDNA detection of *EGFR* mutation status has been recommended by the NCCN and Chinese Society of Clinical Oncology (CSCO) guidelines as complementary means to obtain tumor tissue. But unfortunately, different detection methods show that the sensitivity is only 50.0%–81.8%. Even though next‐generation sequencing has improved the sensitivity of detection of ctDNA *EGFR* mutation status, the detection efficiency and quality cannot be guaranteed due to its high cost and nonstandard operation.^17^, ^18^ In addition, several studies have shown that the efficiencies of EGFR‐TKI in patients with EGFR‐mutant NSCLC can only reach about 70%. Therefore, predictive markers are urgently needed to predict *EGFR* mutation status and EGFR‐TKI efficacy in NSCLC patients.

Several studies have explored the difference between cancer tissue and paracancerous tissues. lncRNA is used to diagnose different cancers, indicating the potential of lncRNA as a diagnostic biomarker. In a study of lung squamous cell carcinoma, Luo *et al*. used a bioinformatics tool to analyze the difference between lncRNA in 450 patients with primary lung squamous cell carcinoma and normal tissues, and showed that AC064853.2, AC090044.2, CTD‐3099C6.9, and KB‐1836B5.4 can be used as biomarkers for the diagnosis of lung squamous cell carcinoma.^19^ Wang *et al*. analyzed the difference between EGFR 19 exon mutation and EGFR wild‐type lncRNA in advanced NSCLC patients by lncRNA microarray and qRT‐PCR. Finally, five lncRNAs were found to play a key role in EGFR 19 exon mutation.^20^ However, few studies have focused on the expression of lncRNA in blood samples associated with *EGFR* mutation status in patients with advanced NSCLC. Recent studies have shown that lncRNAs are present in stable forms in serum, plasma, and other body fluids, independent of endogenous RNase, making them noninvasive suitable biomarkers. In this study, we systematically evaluated the ability of circulating lncRNAs as potential markers for predicting *EGFR* mutation status, EGFR‐TKI efficacy, and monitoring the efficacy of EGFR‐TKI in patients with EGFR‐positive NSCLC. To the best of our knowledge, this study is the first to describe a prospective analysis of circulating lncRNA as a diagnostic or prognostic biomarker for EGFR‐TKI in the treatment of EGFR‐positive NSCLC.

In recent years, several studies have shown that the specificity and sensitivity of pleural effusion detection of ctDNA *EGFR* mutation status are close to, or even higher than, that of tumor tissue. Lee *et al*. used pleural effusion supernatants from 50 patients with lung adenocarcinoma to test for *EGFR* mutation status. That study found that the results of pleural effusion were 100% matched with tumor tissue, and three patients with positive *EGFR* mutations were found among 19 EGFR wild‐type patients. The reason for this may be that pleural effusion overcomes tumor heterogeneity to some extent.^16^ The present study systematically analyzed the differential expression levels of lncRNAs in the pleural effusions of three *EGFR*‐mutant patients and three EGFR wild‐type patients with NSCLC, detected the relative expression levels in pleural effusions and plasma through qRT‐PCR, and comprehensively evaluated the potential of *SCARNA7*, *MALAT1*, and *NONHSAT017369* as prospective markers for *EGFR* mutation status in patients with NSCLC. As observed in a study comparing the clinical efficacy of icotinib and gefitinib in previously treated patients with advanced NSCLC, the therapeutic efficacy of TKIs is better in patients harboring the *EGFR* exon 19 deletion than in patients harboring the p.L858R mutation. Therefore, the identification of potential markers delineating *EGFR* mutant subtypes (mainly the *EGFR* exon 19 deletion and p.L858R point mutation) is also crucial in clinical studies. In this study, three circulating plasma lncRNAs (*SCARNA7*, *MALAT1*, and *NONHSAT017369*) were significantly upregulated in *EGFR*‐mutant patients as compared to EGFR wild‐type patients. Their expression was able to distinguish between *EGFR*‐mutant and wild‐type NSCLC patients with high specificity and sensitivity. In the 3‐lncRNA combination, the PPV and NPV both exceeded 80%. In addition, because EGFR exon 19 deletion has a better therapeutic response to EGFR‐TKI than EGFR21 p.L858R point mutation, further analysis of *EGFR* mutant subtypes shows that plasma MALAT1 is significantly associated with it. Compared with EGFR21 p.L858R point mutation NSCLC patients, MALAT1 was significantly upregulated in plasma of patients with EGFR 19 exon deletion NSCLC.

In recent years, in the NSCLC study, several potential markers have been reported to predict the efficacy of EGFR‐TKI and to monitor the efficacy of EGFR‐TKI. However, most studies are based on tissue or cell line levels, and there are relatively few studies related to serum or plasma levels. Salmon *et al*. used MALDI‐TOF‐MS to test the serum of 13 patients with pre‐NSCLC treated with gefitinib or erlotinib, and an EGFR‐TKI efficacy model with 8 characteristic peaks was established. Then, the groups of 67 patients treated with gefitinib, and 96 treated with erlotinib, and the control group without EGFR‐TKI treatment were tested to predict whether EGFR‐TKI was effective or had a poor efficacy. The model predicted a median survival of 207 days and 92 days for the good and poor patients in the gefitinib group, respectively. The median survival of the good and poor patients in the erlotinib‐treated group was 306 and 107 days, respectively, while the control group could not be used to predict the results.^21^ However, there are few studies on the prediction and monitoring of EGFR‐TKI efficacy at the plasma lncRNA level. To explore the relevance of lncRNA to EGFR‐TKI in patients with *EGFR* mutation‐positive NSCLC, we performed a Kaplan‐Meier analysis of PFS in 59 patients and found that patients with high plasma MALAT1 (≥median) expression after treatment with EGFR‐TKI had longer PFS indicating that plasma MALAT1 has the potential to predict the efficacy of *EGFR* mutation‐positive EGFR‐TKI. Dynamic plasma analysis of patients with EGFR‐TKI response to PR or SD showed a decrease in plasma SCARNA7 and MALAT1 levels, and a lower trend in PR patients than in SD patients. Plasma SCARNA7 and MALAT1 returned to baseline levels as the disease progressed.

To date, no report has described the correlation between *SCARNA7* and *NONHSAT017369* and lung cancer or EGFR signaling. Studies on *SCARNA7* expression levels in other cancers have shown inconsistent results.^22^, ^23^ Most of the previous reports have confirmed that *SCARNA7* is upregulated in cancer tissues, although one report indicated that *SCARNA7* was downregulated in chronic lymphocytic leukemia.^24^ In our study, the plasma *SCARNA7* level was upregulated in *EGFR*‐mutant patients with NSCLC compared to those with wild‐type EGFR. There are few reports on *NONHSAT017369*, and regulatory network analysis found that *NONHSAT017369* can be targeted to IL6R. IL6R is involved in the JAK‐signal transducer and activator of transcription (STAT) signaling pathway, which is commonly known as the signaling pathway downstream of EGFR.^25^, ^26^ Therefore, *NONHSAT017369* may affect EGFR status through STAT activation. *MALAT1* is highly expressed in most tumors; siRNA‐induced knockdown reduces the ability of tumors to invade and metastasize.^7^ *MALAT1* is more highly expressed in metastatic NSCLC than in nonmetastatic NSCLC, making it the first lncRNA confirmed to be able to trigger tumor metastasis and clearly determine the prognosis of patients.^27^ Chen *et al*. found that *MALAT1* plays an important role in tumor progression and the development of resistance to chemotherapeutic drugs.^28^ Yang *et al*. inhibited the activity of gefitinib‐resistant NSCLC and induced apoptosis by regulating PPI to downregulate *MALAT1* and inhibit STAT3 phosphorylation.^29^ Schmidt *et al*. found that STAT3 may play an important role in gefitinib resistance, and *MALAT1* may be a potential target for reversing resistance to EGFR‐TKIs.^30^, ^31^ In this study, *MALAT1* levels were upregulated in *EGFR*‐mutant patients with NSCLC and could distinguish between *EGFR* mutant subtypes. Moreover, serum *MALAT1* decreased during EGFR‐TKI treatment, but gradually increased during the development of drug resistance, which was also consistent with the results of previous studies. This indicated that *MALAT1* can serve not only as a diagnostic marker for *EGFR*‐mutant lung cancer, but also as an important potential marker for monitoring the efficacy of EGFR‐TKI therapy.

This study had several limitations. First, the samples were collected from a single lung cancer center; further external validation of our results requires sample collection from multiple centers. Second, the number of dynamic plasma samples in patients with NSCLC treated with EGFR‐TKIs was relatively small; more samples are required in future work to ensure the reliability of the predictive model. Third, this study failed to identify the expression of lncRNAs in the plasma and tissues of the same individual; future studies require matching plasma and tissue samples from the same individual to verify whether the expression levels of lncRNAs in these samples are consistent. Fourth, cytological experiments on EGFR signal transduction pathways are required to elucidate the specific functions of these lncRNAs. In conclusion, this exploratory study established a group of plasma lncRNAs for predicting *EGFR* mutation status, and this model could serve as a new noninvasive biomarker for the diagnosis of *EGFR*‐mutant patients with NSCLC and an important indicator for predicting the efficacy of EGFR‐TKI therapy.

## Data statement

All data included in this study are available upon request by contacting the corresponding author.

## Disclosure

The authors declare that they have no competing interests.

## Supporting information


**Figure S1** Study overview. This flow chart describes the screening of patients and experimental procedures used in this study.
