# Supplementary figures and images for: Circulating plasma lncRNAs as novel markers of EGFR mutation status and monitors of epidermal growth factor receptor‐tyrosine kinase inhibitor therapy
Source: Thorac Cancer. 2019 Nov 5;11(1):29–40. doi: 10.1111/1759-7714.13216 (PMC6938758; doi:10.1111/1759-7714.13216)

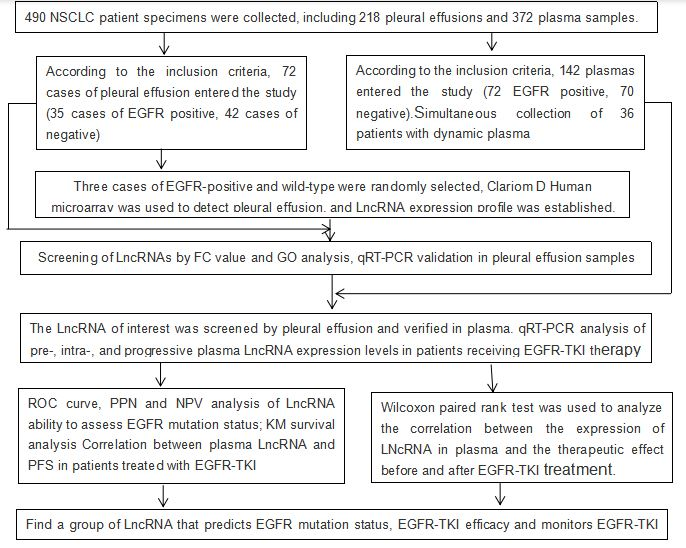

Supplement: Supplementary file 1 — Figure S1 Study overview. This flow chart describes the screening of patients and experimental procedures used in this study. [file TCA-11-29-s001.tif]
